# Electrocardiographie du sportif de haut niveau d’entrainement à Bobo-Dioulasso, Burkina Faso

**DOI:** 10.11604/pamj.2020.36.319.17747

**Published:** 2020-08-21

**Authors:** Somnoma Jean-Baptiste Tougouma, Yibar Kambiré, Aimé Arsène Yaméogo, Samba Sidibé, Jonas Koudougou Kologo, Widouh Benjamin Adolphe Zingue Ouattara, Georges Millogo, Nobila Valentin Yaméogo, Patrice Zabsonré

**Affiliations:** 1 Institut Supérieur des Sciences de la Santé, Université Nazi Boni, Bobo Dioulasso, Burkina Faso,; 2 Service de Cardiologie, Centre Hospitalier Universitaire Sourô Sanou, Bobo Dioulasso, Burkina Faso,; 3 Unité de Formation et de Recherche en Sciences de la Santé, Université de Ouagadougou, Ouagadougou, Burkina Faso,; 4 Service de Cardiologie, Centre Hospitalier Universitaire du Point G, Bamako, Mali

**Keywords:** Cœur, sport de haut niveau, caractéristiques électrocardiographiques, Burkina Faso, Heart, high-performance sport, electrocardiographic features, Burkina Faso

## Abstract

Le sport de haut niveau d’entrainement entraine des modifications électrocardiographiques. Certaines sont bénignes, d’autres sont péjoratifs à l’origine de mort subite. Les auteurs se proposent de décrire les caractéristiques électrocardiographiques de repos chez les sportifs de haut niveau d’entraînement de la ville de Bobo-Dioulasso. Il s’est agi d’une étude transversale descriptive allant d’août 2015 à février 2016 dans le service de cardiologie du CHU Sanou Sourô de Bobo-Dioulasso. Elle a inclus des sportifs de haut niveau d’entrainement âgés de 17 à 35 ans ayant au moins huit heures d’entrainement par semaine depuis plus de six mois quel que soit le type de sport. Deux cents sportifs de haut niveau d’entrainement de 4 disciplines sportives ont été inclus. L’âge médian des athlètes était de 24 ans (IIQ: 21-27). L’ancienneté médiane de la pratique du sport à haut niveau d’entrainement était de 6 ans (IIQ: 4-8) et la durée médiane des séances d’entraînement hebdomadaire de 10 heures (IIQ: 10-10). Seulement 4% des athlètes avaient déjà réalisé un électrocardiogramme (ECG). L’ECG présentait des anomalies dans 90,5% et la bradycardie sinusale était l’anomalie la plus fréquente rapportée dans 72,5% des cas. L’hypertrophie ventriculaire gauche et celle atriale gauche étaient rapportées respectivement dans 44% et 34,5%. Le syndrome de repolarisation précoce était retrouvé dans 47% des cas. La pratique du sport de haut niveau d’entraînement peut occasionner des modifications électriques chez l’athlète. Celles-ci nécessitent d’être connus par les praticiens afin de les différencier de la pathologie cardiaque.

## Abstract (en)

High-performance sport results in electrocardiographic changes. Some are benign, other can cause sudden death. The purpose of this study is to describe the features of electrocardiogram at rest in elite athletes living in Bobo-Dioulasso. We conducted a cross-sectional descriptive study in the Department of Cardiology at the Souro Sanou University Hospital in Bobo-Dioulasso from August 2015 to February 2016. Elite athletes aged 17 to 35 years who had been training at least eight hours per week for more than six months, regardless of the type of sport, were enrolled. Two hundred elite athletes from four different sporting disciplines were included. The average age of athletes was 24 years (IIQ: 21-27). The median seniority in sport practice was 6 years (IIQ: 4-8) and the median duration of weekly training was 10 hours (IIQ: 10-10). Only 4% of the athletes had already undergone electrocardiogram. ECG showed abnormalities in 90.5% of cases and sinus bradycardia was the most common abnormality in 72.5% of cases. Left ventricular hypertrophy and left-atrial dilatation were reported in 44% and 34.5% respectively. Early repolarization syndrome was found in 47% of cases. In athletes, high-performance sport can result in electrical modifications. Practitioners need to know them in order to differentiate them from heart disease.

## Introduction

Bien que décrit de longue date [1], le cœur d’athlète constitue un sujet d’actualité en médecine du sport et en médecine générale comme l’illustrent les dernières recommandations des sociétés savantes, européenne et américaine. Il a bénéficié des méthodes d’exploration récentes et en particulier, des méthodes non invasives que sont l’électrocardiogramme (ECG) et l’échocardiographie Doppler [2]. Ces particularités du cœur de l’athlète méritent d’être connues des praticiens. Celles-ci peuvent parfois poser des problèmes de diagnostic différentiel avec des pathologies cardiaques à risque, notamment la cardiomyopathie hypertrophique (CMH), qui est considérée comme la plus grande pourvoyeuse de mort subite de causes cardiaques chez les sportifs de moins de 35 ans [3]. La survenue de la mort subite au cours du sport révèle un cardiaque ignoré ou qui a été ignoré, d’où l’intérêt de la visite médicale de non contre-indication (VNCI) à la pratique sportive en loisir ou en compétition. Plusieurs études ont été réalisées dans le monde sur les particularités du cœur de sportif de haut niveau [1-3]. Mais il existe très peu de travaux portant sur les spécificités chez les sportifs noirs africains. Au Burkina Faso, aucun travail à notre connaissance n’a encore porté sur les anomalies électrocardiographiques du cœur d’athlète de haut niveau d’entrainement. Cette absence de données malgré la présence du sport burkinabé au niveau de l’élite africaine est un vide à combler. C’est pourquoi, nous avons décidé de mener une étude transversale descriptive dans la ville de Bobo-Dioulasso afin d’étudier les anomalies électrocardiographiques de repos chez les sportifs de haut niveau d’entraînement.

## Méthodes

**Type et période de l’étude:** il s’est agi d’une étude descriptive transversale réalisée d’août 2015 à février 2016 dans le service de cardiologie du Centre Hospitalier Universitaire Sourô Sanou (CHUSS) de Bobo-Dioulasso (Burkina Faso). La ville de Bobo-Dioulasso compte beaucoup de champions nationaux dans les différentes disciplines avec quelques médaillés au plan africain.

**Population de l’étude et critères d’inclusion:** il s’agissait des sportifs de haut niveau d’entrainement âgés de 17 à 35 ans ayant au moins huit heures d’entrainement par semaine depuis plus de six mois quel que soit le type de sport. Les critères d’inclusion étaient de pratiquer le sport dans un cadre réglementé (clubs) et d’accepter de prendre part à l’étude. Ont été exclus de l’étude, les sportifs présentant une pathologie cardiaque; ceux ayant un arrêt pour maladie depuis plus de 3 mois ou ayant un handicap.

**Méthodes et techniques de collecte de données:** les sportifs ont été mobilisés pour l’étude via les responsables et entraîneurs des clubs. L’ancienneté du sportif dans le club, la régularité, l’assiduité aux séances d’entraînements et les arrêts pour maladies ont été collectées auprès de ces dirigeants. Les sportifs identifiés étaient accueillis au service de cardiologie du CHUSS dans une salle réservée à cet effet pour le processus d’information et de consentement éclairé pour la participation à l’étude. Après la signature du consentement, chaque enquêté bénéficiait d’un interrogatoire et d’un examen clinique complet notamment cardiovasculaire et d’un enregistrement électrocardiographique de surface et de repos (12 dérivations) à l’aide d’un appareil de marque HELLIGE EK53. A l’interrogatoire les données collectées étaient: les caractéristiques sociodémographiques (âge, sexe, profession, niveau d’étude), les données sur l’activité sportive (discipline, ancienneté, volume horaire d’entraînements par semaine, nombre de participation aux compétitions nationales et internationales). Les facteurs de risque cardiovasculaire: hypertension artérielle (HTA) personnelle ou familiale, surpoids, obésité, tabagisme actif ou passif, diabète, dyslipidémie, consommation de thé/café, d’alcool, de stupéfiants et /ou anabolisants; l’hérédité cardiovasculaire (HTA, coronaropathie/accident ou maladie cardiaque et antécédent de mort subite familiale avant 50 ans). Les signes fonctionnels liés à l’effort (syncope ou lipothymie, gène ou douleur thoracique, palpitations ou irrégularités du rythme cardiaque, essoufflement/ou fatigue inhabituelle). Les données collectées lors de l’examen clinique étaient: les constantes anthropométriques et cliniques (poids, taille, index de masse corporelle (IMC), surface corporelle, fréquence cardiaque, pouls pression artérielle après 15 minutes de repos); les signes physiques (rythme cardiaque, souffles cardiaques, pouls fémoraux, varices; la carie dentaire). A l’ECG les variables suivantes étaient recueillies: fréquence cardiaque de repos, rythme, durée et amplitude de l’onde P, durée de l’espace PR, durée du complexe QRS, l’axe des QRS, index de Sokolov-Lyon, le QT corrigé ainsi que l’aspect de la repolarisation. Les résultats des enregistrements électrocardiographiques étaient validés par deux cardiologues.

**Taille de l’échantillon:** l’échantillonnage a tenu compte des différents groupes ou disciplines sportives pratiquées dans la ville de Bobo. Sur les 1811 sportifs licenciés juniors et seniors des 101 clubs sportifs de 18 disciplines sportives, 394 répondaient au critère de sportif de haut niveau d’entraînement. Ils provenaient de quatre disciplines que sont : football (92,5%); athlétisme (2,5%); cyclisme (2,5%); judo (2,5%). Nous avons utilisé OPENEPI [4] pour calculer la taille minimale de l’échantillon. La prévalence attendue du cœur d’athlète dans la population d’étude fixée à 50%; la précision souhaitée à 5% et z_1-α/2_= 1.96. A partir d’une enquête de terrain, la population source a été estimée à 394 sportifs sur la base des statistiques de la Direction Régionale des Sports/Hauts bassins, des ligues et districts sportifs. Cela donnait une taille minimale de l’échantillon calculée de 195 sportifs.

**Analyse des données:** les données recueillies sur une fiche individuelle ont été saisies et analysées à l’aide des logiciels Epi Data version 3.1 et Stata version 12.0. L’analyse a consisté à la production de statistiques descriptives (médiane ou proportion) de la population d’étude.

**Considérations éthiques:** la participation à notre étude était conditionnée par la signature d’un consentement éclairé. Le refus de prendre part à l’étude n’entrainait aucun préjudice. La confidentialité a été respectée et le traitement des données a été anonyme. La participation à l’étude n’entrainait aucun frais pour les sujets inclus.

## Résultats

Au total 200 athlètes de haut niveau d’entrainement ont été inclus dans notre étude. L’âge médian était de 24 ans (IIQ: 21-27). La quasi-totalité de la population étudiée était constituée d’hommes (98,5%) et 18% étaient des élèves ou étudiants (Tableau 1).

**Tableau 1:** caractéristiques sociodémographiques des 200 sportifs de haut niveau d’entrainement, Bobo-Dioulasso, 2016

Caractéristiques	N=200
Age, médiane (IIQ)	24 (21-27)
Age [20-29] (%)	80
Sexe masculin (%)	98,5
Activité professionnelle (%)	48,5
Elèves et étudiants (%)	18
IMC, médiane (IIQ)	22,5 (21,2-23,7)
Surpoids (%)	8,5
**Niveau d´étude (%)**	
Aucun	4,5
Primaire	16
Secondaire	77
Supérieur	2,5

**Les données de la pratique sportive:** la répartition selon la discipline sportive était de 185 (92,5%) pour le football, 5 (2,5%) pour le judo, 5 (2,5%) pour l’athlétisme et 5 (2,5%) pour le cyclisme. L’ancienneté médiane de la pratique du sport de haut niveau d’entrainement était de 6 ans (IIQ: 4-8). La durée médiane des séances d’entraînement hebdomadaire était de 10 heures (IIQ: 10-10). Le nombre médian de participations aux compétitions nationales était de 13 (IIQ: 4-18).

**Caractéristiques cliniques:** il ressortait que 4,7% des athlètes avaient déjà ressentis des malaises d’effort et 2% de douleurs thoraciques. Quarante virgule cinq pourcent (40,5%) avaient des antécédents familiaux d’HTA et 1,5 avaient des chiffres tensionnels anormaux. Aucun antécédent de mort subite familiale n’a été rapporté dans notre étude. Seulement 8 (4%) sportifs avaient déjà réalisé un ECG dans leur carrière de sportif de haut niveau. La consommation de tabac, d’alcool, de thé/café, de médicaments étaient rapportée respectivement dans 3,5%, 28%, 99%, 28,5%. La bradycardie était présente chez 72% des athlètes (Tableau 2).

**Tableau 2:** caractéristiques cliniques des 200 sportifs de haut niveau d’entrainement, Bobo-Dioulasso, 2016

Caractéristiques	N= 200
Antécédents de symptômes d’effort %	10,5
Malaises %	4,7
Douleurs thoraciques %	2
Dyspnée %	3,8
Tabagisme actif %	3,5
Antécédents mort subite %	0
Antécédents familiaux d’HTA %	40,5
Antécédents familiaux de diabète %	6,5
Consommation occasionnelle d’alcool %	7,3
Consommation de thé et/ou café %	99
Consommation de stupéfiants %	0
Consommation de médicaments %	28,5
CaC1000 (acide ascorbique) %	27
Gurosan (acide ascorbique + glucuronamide + caféine) %	1,5
Boisson énergisante %	3
Red Bull	1,5
XXL	1,5
Fréquence cardiaque, médiane (IIQ)	54,5 (IIQ : 50-60)
Bradycardie (FC<60/minute)%	72
PAS, moyenne	116,29±8,28 [91,5-145]
PAD, moyenne	71,86±7,73 [56-93,5]
HTA de grade 1 %	1,5

Red Bull (saccharose, glucose, acide ascorbique, taurine, cafeine, niacine, vitamines B5,B6,B12) XXL (saccharose, glucuronolactone, mélange de vitamines, choline, inositol, caféine,taurine, gingeng...)

**Anomalies électrocardiographiques:** l’ECG réalisé au cours de notre étude présentait des anomalies dans 90,5% des cas. La fréquence cardiaque moyenne était de 54,52±8,36 (37 et 83). La bradycardie sinusale était l’anomalie la plus fréquente rapportée dans 72,5% des cas. Onze cas (5,5%) d’arythmie sinusale respiratoire et un cas (0,05%) d’extrasystole ventriculaire ont été rapportés. L’axe était gauche chez sept sportifs (3,5%). Aucun cas d’axe droit n’a été rapporté. L’espace PR médian était de 180ms (IIQ: 160-200). Un cas (0,05%) d’espace PR court avec onde delta associée évocateur d’une préexcitation ventriculaire a été rapporté. Le bloc auriculo ventriculaire (BAV) du 2^e^ degré Mobitz 1 type Lucianni Wencheback était présente chez trois (1,5%) athlètes. L’hypertrophie auriculaire gauche était retrouvée chez 69 sportifs (34,5%) et l’hypertrophie auriculaire droite chez 11 des sportifs (5,5%). On retrouvait 88 cas (44%) d’hypertrophie ventriculaire gauche (HVG) avec un indice médian de Sokolov-Lyon à 40mm (IIQ: 37-44) et six cas (3%) d’hypertrophie ventriculaire droite. Quarante-six cas (23%) de bloc de branche droit incomplet et sept (3,5%) cas d’hémi-bloc antérieur gauche ont été rapportés. Aucun cas de bloc de branche gauche complet ou incomplet, et d’hémi-bloc postérieur n’a été rapporté. Le syndrome de repolarisation précoce était retrouvé dans 94 cas (47%) le plus souvent dans le territoire antéro-septal et apical. La valeur médiane du QTc était de 387ms (IIQ: 368-409). On notait 4 cas (2%) d’allongement du QTc (Tableau 3).

**Tableau 3:** anomalies électrocardiographiques des 200 sportifs de haut niveau d’entrainement, Bobo-Dioulasso, 2016

Anomalies électrocardiographiques	N(%)
HAG	69(34,5)
HAD	11(5,5)
Axe gauche	7(3,5)
Onde T ample	11(12,09)
Onde Q anormale ou QS	0
BBD	46(23)
BBG	0
R/S>1	0
Sous ST, T<0	14(15,28)
QTc>0,44	4(2)
ESV	1(0,5)
TSV, Flutter, FA	0
Préxcitation ventriculaire	1(0,5)
BAV1	35(17,5)
BAV2	3(1,5)
BAV3	0
Bradycardie sinusale <40bpm	2(1)

## Discussion

Une limite importante de notre travail était la difficulté d’attribuer les anomalies observées dans notre étude au seul fait du sport en l’absence d’ECG de base en début de pratique du sport et de l’absence d’un groupe témoin. Cependant, les similitudes des données avec d’autres études nous permettent de discuter raisonnablement l’impact du sport même si le niveau de responsabilité de celui-ci ne peut être précisé. L’ECG de repos est le premier examen du bilan paraclinique de l’athlète. Dans notre étude, seulement 4% des sportifs avaient déjà réalisé cet examen de routine dans leur carrière d’athlète. Pourtant, il est connu que cet examen simple permet la détection d’au moins 60% des pathologies cardiovasculaires de l’athlète [5-7]. Cette situation témoigne de l’absence de la visite de non contre-indication à la pratique du sport même de haut niveau dans notre contexte burkinabé. Elle interpelle les responsables à tous les niveaux de la pratique du sport au Burkina Faso. Un cadre réglementaire devrait être mis en place pour pallier à cette insuffisance en matière de prévention des accidents cardiaques liés au sport. Le cœur du sportif de haut niveau peut-être strictement normal sur le plan électrique, ce qui ne peut remettre en cause son niveau d’entrainement [8]. Dans notre série, 55,5% des sportifs avaient un ECG strictement normal ou avec anomalies mineures. Pelliccia *et al*. [9], en Italie, sur une étude de 1005 athlètes pratiquant 38 disciplines sportives différentes avec une moyenne d’âge similaire à la nôtre, rapportait une proportion semblable à celle retrouvée dans notre étude (60%). Dans notre série, la bradycardie sinusale était la plus fréquente des modifications communément associées au cœur d’athlète (72,5%). Cette proportion est sensiblement égale à celle de Gaëlle K *et al*. [5] chez les footballeurs Japonais qui était de 69,1% et proche de celles rapportées par Balady *et al*. [10] et Wilson *et al*. [7] qui étaient respectivement de 77% et 80%. La bradycardie est le résultat d’une adaptation physiologique du système nerveux autonome en rapport avec une hypertonie vagale. Elle est courante chez l’athlète et dépend du type de sport et du niveau d’entrainement/compétition [6,10,11]. Les BAV sont communément décrits chez les athlètes entrainés. Dans notre série, le BAV de 1^er^ degré a été noté dans 17,5% des cas, supérieur au taux de 9,7% retrouvé par Ba *et al*. [12]. Nos résultats sont proches de ceux rapportés par Wilson *et al*. [7] et Crouse *et al*. [13] qui étaient respectivement de 13% et 14,8%. Le BAV 2 type Mobitz 1 a été retrouvé chez trois (1,5%) footballeurs. Ce résultat est similaire à celui obtenu par Lahady *et al*. [14] et Ba *et al*. [12] qui retrouvaient chacun deux cas dans leur série respective. Le ralentissement de la conduction atrio-ventriculaire et les blocs atrio-ventriculaires sont liés à une augmentation du tonus parasympathique associée ou non à une diminution du tonus sympathique [15]. La prévalence de l’HVG dans notre série a été de 44%, supérieure aux taux respectifs de 25,80%, 26,8% et 36,3% rapportés par Siransy *et al*. [16], Wilson *et al*. [7] et Ondze *et al*. [17] mais inférieure à celles retrouvées dans les séries de Ba *et al*. [12] et Di Paolo *et al*. [18] qui étaient de 85,44% et 89%. Ces différences sont certainement en rapport avec les critères utilisés pour définir l’HVG électrique chez le sportif. En effet, les critères de Sokolow-Lyon, les plus couramment utilisés, tendent à majorer la fréquence des HVG électriques chez le sportif contrairement à d’autres critères souvent utilisés par certains auteurs. Le syndrome de repolarisation précoce était de 47% chez nos sportifs, similaire à la proportion de 49,51% retrouvée par Ba *et al*. [12] chez les footballeurs sénégalais. Sharma *et al*. [19] a trouvé une fréquence proche de la nôtre de l’ordre de 43%. Cette repolarisation précoce était plus fréquemment rencontrée dans les dérivations V3, V4 et V5. Tel était également le constat fait par Ba *et al*. [12] dans sa série. Il est communément admis que la repolarisation précoce est plus répandue chez les sportifs [18,20] et liée à une hypervagotonie. Bien que fréquente, elle reste une anomalie bénigne qui ne doit pas être banalisée car certains auteurs lui ont attribué un risque plus élevé de fibrillation ventriculaire [21].

## Conclusion

Les anomalies électrocardiographies des athlètes de haut niveau d’entraînement sont dominées par la bradycardie sinusale, l’hypertrophie ventriculaire gauche et le syndrome de repolarisation précoce dans notre étude. L’électrocardiogramme, examen simple d’acquisition, malgré sa forte valeur prédictive positive d’évènements cardiovasculaires n’est pas encore de routine chez les athlètes de haut niveau d’entrainement au Burkina Faso. Des efforts devraient être faits pour instaurer de façon systématique la visite de non contre-indication à la pratique du sport de haut niveau avec la réalisation d’un électrocardiogramme. Cela permettra la détection des cardiopathies à risque de mort subite chez nos athlètes comme le recommandent les sociétés savantes.

### Etat des connaissances actuelle sur le sujet

- Le cœur d’athlète est un sujet d’actualité;
- La survenue de la mort subite au cours du sport révèle un cardiaque ignoré ou qui a été ignoré;
- Les anomalies électrocardiographiques sont de nos jours bien décrits.

### Contribution de notre étude à la connaissance

- La prévalence des anomalies électrocardiographiques chez le sportif de haut niveau d’entrainement est connue au Burkina Faso;
- L’ECG sera systématiquement réalisé à la visite de non contre-indication à la pratique du sport de haut niveau à Bobo-Dioulasso.
